# Early and late morbidity and mortality and life expectancy following thoracoscopic talc insufflation for control of malignant pleural effusions: a review of 400 cases

**DOI:** 10.1186/1749-8090-5-27

**Published:** 2010-04-19

**Authors:** Nikolaos Barbetakis, Christos Asteriou, Fani Papadopoulou, Georgios Samanidis, Dimitrios Paliouras, Athanassios Kleontas, Konstantina Lyriti, Ioannis Katsikas, Christodoulos Tsilikas

**Affiliations:** 1 Thoracic Surgery Department, Theagenio Cancer Hospital, A. Simeonidi 2, Thessaloniki, 54007, Greece; 2 Thoracic Anesthesia Department, Theagenio Cancer Hospital, A. Simeonidi 2, Thessaloniki, 54007, Greece

## Abstract

**Background:**

Malignant pleural effusion is a common sequelae in patients with certain malignancies. It represents a terminal condition with short median survival (in terms of months) and the goal is palliation. Aim of our study is to analyze morbidity, mortality and life expectancy following videothoracoscopic talc poudrage.

**Materials and methods:**

From September 2004 to October 2009, 400 patients underwent video-assisted thoracic surgery (VATS) for malignant pleural effusion. The conditions of patients were assessed and graded before and after treatment concerning morbidity, mortality, success rate of pleurodesis and median survival.

**Results:**

The median duration of follow up was 40 months (range 4-61 months). All patients demonstrated notable improvement in dyspnea. Intraoperative mortality was zero. The procedure was well tolerated and no significant adverse effects were observed. In hospital mortality was 2% and the pleurodesis success rate was 85%. A poor Karnofsky Performance Status and delay between diagnosis of pleural effusion and pleurodesis were statistically significant factors for in-hospital mortality. The best survival was seen in breast cancer, followed by ovarian cancer, lymphoma and pleural mesothelioma.

**Conclusions:**

Video-assisted thoracoscopic talc poudrage is an effective and safe procedure that yields a high rate of successful pleurodesis and achieves long-term control with marked dyspnea decrease.

## Introduction

Pleural effusions are a common and devastating complication of advancedmalignancies. These effusions most commonly occur with lung, breast, ovarian cancer and lymphoma, with breast and lung malignancies alone accounting for approximately of 75% of these effusions [1].

In patients who develop progressive pleural effusions producing dyspnea and cough, quality of life is affected. Patients with symptomatic effusions may benefit from pleurodesis to relieve dyspnea and to prevent reaccumulation of pleural fluid.

The purpose of this study was to determine the long term efficacy and safety of pleurodesis by thoracoscopic talc poudrage in malignant pleural effusions.

## Materials and methods

All patients with symptomatic malignant pleural effusion referred to the Thoracic Surgery service of Theagenio Cancer Hospital for thoracoscopic pleurodesis were eligible to participate in this study. Inclusion in the study required documentation of a malignant pleural effusion and good general condition (capability to care for themselves). The diagnosis of pleural carcinomatosis was established by positive pleural fluid cytology on thoracentesis or evidence of malignancy on pleural biopsy prior to referral. Patients with significant loculated effusions or trapped lung after drainage were excluded from the study. All patients underwent preoperative bronchoscopy to exclude endobronchial obstruction and chest computed tomography scan.

Thoracoscopy was performed under general anesthesia in all patients. A 10-mm camera port and one or two instrumentation ports were inserted. We used a zero grade optical camera to assess the pleura and the lung surface. The pleural effusion was carefully aspirated and fibrinous adhesions were divided with diathermy. At least four different biopsy specimens were obtained from abnormal areas and a frozen section was performed. The degree of lung expansion was ascertained with sustained positive pressure ventilation (20-30 cm H2O). Pleurodesis was performed by thoracoscopic insufflation of sterile asbestos-free talc powder (6 gr) in all patients regardless the extent of disease. At the end of the procedure one chest tube was left in situ. The drain was removed when the volume collected remained under 200 ml for two consecutive days.

Morbidity included all complications occurring during hospitalization only. In-hospital mortality included both those patients who died within first 30 days and those who died later but during the same hospitalization. Three-month mortality included those patients who died within 90 days after surgery. Follow up was obtained by periodical clinical examination combined with chest radiography and/or thoracic ultrasound. The failure of pleurodesis was defined by a need for repeat thoracentesis or tube thoracostomy to drain a recurrent pleural effusion during the 3 months after pleurodesis.

Univariate analysis was used for continuous variables associated with In-Hospital mortality and 3-Month Mortality. Data are mean ± standard deviation. Differences were considered significant with p values of p < 0.05.

The study was approved by the Investigational Review Board at Theagenio Cancer Hospital and informed consent was obtained from all eligible patients.

## Results

From September 2004 to October 2009, 400 patients underwent video-assisted thoracic surgery (VATS) for recurrent malignant pleural effusion. The characteristics of patients and the underlying malignant disease are shown in Tables 1 and 2 respectively.

**Table 1:** Characteristics of the study population (n: 400).

CHARACTERISTICS	Median (range)
Age (y)	63.2 (21-78)

Sex (male/female)	261/139

Weight loss > 5 kg	84 pts

Karnofsky Status	50 (10-90)

**Table 2:** Pathology of 400 malignant pleural effusions.

PATIENTS		
**MALIGNANCY**	**No**	**%**

Non small cell lung cancer	166	41.5

Breast cancer	97	24.2

Ovarian cancer	24	6

Uterine cancer	16	4

Colon cancer	16	4

Sarcoma	14	3.5

Gastric cancer	13	3.2

Lymphoma	13	3.2

Small cell lung cancer	10	2.5

Pleural mesothelioma	10	2.5

Melanoma	7	1.7

Oesophageal cancer	4	1

Testicular cancer	4	1

Hepatocellular cancer	3	0.7

Parotid gland cancer	2	0.5

Gallbladder cancer	1	0.2

The average duration of VATS talc pleurodesis combined with pleural or lung biopsies was 26 minutes (± 6 min). The conversion rate to thoracotomy was 0%. No operative deaths and no intraoperative major complications were occurred. Three hundred and ninety three patients were weaned from mechanical ventilation in the operating theatre. Seven patients were transferred intubated to the intensive care unit.

Postoperative complications occurred in 66 patients and are shown in Table 3. Our rates were rather low, with the only exception of transient air leak (9%). This complication could be attributed not only to lung or visceral pleura biopsies but also to the rupture of necrotic tumor nodules at the moment of lung reexpansion. Persistent air leak was managed successfully through a carefully applied progressive suction for usually 48 hours with an exception of one case. Under no circumstances was talc-induced ARDS observed.

**Table 3:** Complications in 400 patients with malignant pleural effusion and thoracoscopic talc poudrage performed.

COMPLICATIONS	N	(%)
Prolonged air leak	36	(9)

Persistent pain	31	(7.7)

Subcutaneous emphysema	30	(7.5)

Acute respiratory failure	7	(1.7)

Bleeding	4	(1)

Lung laceration	4	(1)

Wound infection	4	(1)

Persistent pleural space	3	(0.7)

Tumor recurrence at port site	3	(0.7)

Myocardial infarction	3	(0.7)

Empyema thoracis	2	(0.5)

Pulmonary embolism	2	(0.5)

Reexpansion pulmonary edema	1	(0.2)

The average duration of chest drainage was 6 days (range: 2 - 10). The average duration of postoperative hospitalization was 7 days (range: 4 - 9) for the patients without postoperative complications versus 16 days (range: 7 - 40) for the patients with postoperative complications.

Eight patient deaths (8/400, 2%) occurred during hospitalization. The cause of death was pneumonia in 3 patients, pulmonary embolism in 2 patients, myocardial infarction in 2 patients and septic shock in 1 patient. The death rate within 3 months after pleurodesis was 15/400 (3.7%). Factors adversely affecting in hospital mortality and 3-month mortality included age, Karnofsky Performance Status and delay between diagnosis of pleural effusion and pleurodesis. The last two factors were found to be statistically significant (Table 4).

**Table 4:** Continuous variables associated with In-Hospital mortality and 3-Month Mortality in Univariate Analysis.

	In hospital mortality		3-month mortality	
**VARIABLE**	**No (n:392)**	**Yes (n:8)**	**No (n:385)**	**Yes (n:15)**

Age (years)	61 ± 16	69 ± 14	60 ± 14	71 ± 13

K.P.S.	74 ± 14	60 ± 18*	76 ± 18	60 ± 16*

Delay between diagnosis and pleurodesis (days)	40 ± 16	66 ± 36*	45 ± 16	78 ± 60*

Data are mean ± standard deviation

* p < 0.05.

The median duration of follow up was 40 months (range 4-61 months). The post-pleurodesis average survival according to primary malignancy is shown in Table 5. The best survival was seen in breast cancer, followed by ovarian cancer, lymphoma and pleural mesothelioma.

**Table 5:** Average survival following pleurodesis according to primary malignancy.

PATIENTS	
**MALIGNANCY**	**Average survival (months)**

Breast cancer	13.6

Ovarian cancer	13.1

Lymphoma	10.2

Pleural mesothelioma	9.6

Colon cancer	8.4

Melanoma	7.8

Parotid gland cancer	7.0

Non small cell lung cancer	6.7

Small cell lung cancer	6.6

Uterine cancer	6.4

Sarcoma	6.4

Testicular cancer	6.0

Gastric cancer	4.9

Oesophageal cancer	4.0

Hepatocellular cancer	3.4

Gallbladder cancer	3.0

Three hundred and forty patients (340/400 - 85%) had a lasting pleural symphysis until death or the date of last follow up. The exact relation between primary malignancy and success rate is indicated in Table 6.

**Table 6:** Success rate, 3 and 6 months following thoracoscopic talc pleurodesis.

PATIENTS				
**MALIGNANCY**	**Success Rate after 3 months (340/400) [85%]**		**Success Rate after 6 months (328/400) [82%]**	

Non small cell lung cancer	136/166	[81.9]	130/166	[78.3]

Breast cancer	94/97	[96.9]	91/97	[93.8]

Ovarian cancer	22/24	[91.6]	22/24	[91.6]

Uterine cancer	13/16	[81.2]	12/16	[75.0]

Colon cancer	13/16	[81.2]	12/16	[75.0]

Sarcoma	11/14	[78.5]	10/14	[71.4]

Gastric cancer	10/13	[76.9]	10/13	[76.9]

Lymphoma	11/13	[84.6]	11/13	[84.6]

Small cell lung cancer	8/10	[80.0]	8/10	[80.0]

Pleural mesothelioma	8/10	[80.0]	8/10	[80.0]

Melanoma	5/7	[71.4]	5/7	[71.4]

Oesophageal cancer	2/4	[50.0]	2/4	[50.0]

Testicular cancer	3/4	[75.0]	3/4	[75.0]

Hepatocellular cancer	2/3	[66.6]	2/3	[66.6]

Parotid gland cancer	1/2	[50.0]	1/2	[50.0]

Gallbladder cancer	1/1	[100.0]	Death with no recurrence	

## Discussion

Malignant pleural effusions are one of the leading causes of recurrent pleural effusions worldwide, with an estimated annual incidence of 150.000 cases in the United States [2]. Dyspnea that arises from pleural effusion impacts considerably the quality of life. Thoracentesis is an essential first step but may only provide temporary relief and can be associated either with the recurrence of pleural effusion (90% of patients will develop recurrence of effusion within 30 days) or to iatrogenic pneumothorax, pleural fluid loculation or contamination with subsequent empyema [3]. Simple chest tube drainage is also associated with recurrence of pleural effusion (80% of patients within 30 days after removal of the tube) [4]. Chest tube drainage and chemical pleurodesis is the gold standard of care for malignant pleural effusions. Tetracycline the agent used most commonly in the past, is no longer commercially available. Many other chemotherapeutic agents such as doxorubicin, cisplatin and cytarabine combination, etoposide, fluorouracil, mitomycin, mitoxantrone have been used for sclerotherapy. In addition radioactive isotopes, corynebacterium parvum, interferon and recombinant interleukin-2 have been instilled in the pleural space for treatment of malignant pleural effusions. Response rate has been variable and less than optimal [5].

Silver nitrate 0.5% has proved to be an efficient alternative to tetracycline derivatives and talc for inducing pleurodesis in experimental studies. Its efficacy has also been proved in clinical studies. In patients with malignant pleural effusions who received 0.5% silver nitrate or 5 g of talc "slurry," silver nitrate was more effective in inducing pleurodesis after a 1-month evaluation (95.6% vs 87.5%) and had no significant adverse systemic effects [6].

Over the last decade, indwelling pleural catheter drainage has established itself as a less expensive, minimally invasive, and palliative modality for the management of malignant pleural effusions. Dozens of recent publications on its utility and efficacy for the long-term management of these effusions have increased its popularity as an alternative to conventional modalities [7].

Talc ([Mg_3_Si_4_]O_10 _[OH]_2_) is a trilayered magnesium sheet silicate. Preparations historically have had some minimal associated impurities, most notably asbestos. Talc can be used during thoracoscopy or thoracotomy, or as a slurry via thoracostomy. Chambers using talc slurry in 1958, was the first to utilize talc for the treatment of malignant pleural effusions [8].

Video-assisted thoracoscopy with talc poudrage has replaced conventional instillation of talc slurry through tube thoracostomy as the painless procedure of choice to achieve pleurodesis. It also offers the advantage of complete evacuation of the pleural cavity and visualization of the pleural surface allowing multiple biopsies to be performed with very high accuracy. Futhermore, adhesions may be broken up with confirmation of complete lung reexpansion. This method also permits the distribution of talc in a uniform manner, even in the most inaccessible areas with acceptable morbidity as shown in our study. On the other hand, other investigators advocate that talc slurry instillation is the procedure of choice for patients with symptomatic malignant pleural effusions without trapped lungs due to cost-effectiveness [9]. In our institution, chemical pleurodesis by instillation of asbestos-free talc is strongly recommended in patients with poor Karnofsky Performance Status with an expected median survival of less than 3 months.

In our series in-hospital mortality was 2%, approximately the same with other series [10,11]. The mortality rate within 3 months was 3.7%, with Karnofsky Performance Status and delay between diagnosis of pleural effusion and pleurodesis to play a statistically significant role. According to the international literature there is a credible possibility that aggressive diseases are responsible for a rapid and plentiful recurrence of pleural effusion and limited life expectancy. Sahn and Good showed that this type of pleural effusion correlated with a pH of 7.28 or less or with a lower glucose concentration [12]. Rodriguez-Panadero and Lopez-Mejias also suggested that the extent of pleural lesions detected during thoracoscopy was closely related to both glucose and hydrogen ion concentrations in pleural fluid and that duration of survival was inversely related to the extent of carcinomatous involvement of the pleura [13]. These pleural fluid characteristics were not examined in our study.

The failure rate of videothoracoscopic talc pleurodesis was 15% and is higher compared to other series reported, with a range of failure rate from 0% to 7% [11,14,15]. The possible explanation is that it is difficult to compare our data with other series; the characteristics of our patients are different as well as the primary malignancies.

There are however, potential limitations to our study. The retrospective study design could have introduced systemic bias, including patients who were unavailable for follow up. This problem was eliminated by using data that were derived from a 90- and 180-day period with complete outcome information for statistical analysis. Furthermore the quality of life was not documented in the months following the procedure. Successful pleurodesis is linked to marked improvement in dyspnea. However the patient benefit regarding quality of life still remains to be elucidated.

## Conclusions

In conclusion videothoracoscopic talc poudrage represents a safe and reliable method to obtain pleurodesis in patients with malignant recurrent pleural effusion non-responding to corticosteroid therapy and or to chemotherapy. The long-term results show a high successful rate. A more effective pleurodesis is likely, if videothoracoscopic talc poudrage is performed early after the diagnosis and the lung is free to reexpand.

## Competing interests

The authors declare that they have no competing interests.

## Authors' contributions

NB conceived of the study and participated in its design and coordination. CA participated in the design of the study and performed the statistical analysis. FP, GS, DP, AK, KL and IK took part in the care of the patients and contributed equally in carrying out the medical literature. CT had the supervision of this report. All authors approved the final manuscript.
